# Individual alpha frequency tACS modifies the detection of space–time optical illusion

**DOI:** 10.1007/s00221-025-07158-w

**Published:** 2025-09-09

**Authors:** Francesco Neri, Vincenzo Catrambone, Alessandra Cinti, Adriano Scoccia, Alberto Benelli, Sara Romanella, Laetitia Grabot, Gaetano Valenza, Carmelo Luca Smeralda, Emiliano Santarnecchi, Virginie van Wassenhove, Simone Rossi

**Affiliations:** 1 Siena Brain Investigation and Neuromodulation Lab (Si-BIN Lab), Department of Medicine, Surgery and Neuroscience, Neurology and Clinical Neurophysiology Section, University of Siena, Siena, Italy; 2 Oto-Neuro-Tech Conjoined Lab, Policlinico Le Scotte, University of Siena, Siena, Italy; 3 Department of Information Engineering, University of Pisa, Bioengineering & Robotics Research Center “E. Piaggio”, Pisa, Italy; 4 Cognitive Neuroimaging Unit, CEA, DRF/Joliot, NeuroSpin, INSERM, Université Paris-Saclay, 91191 Gif-sur-Yvette, France; 5 Department for Cognitive Neuroscience, Faculty of Biology & Cognitive Interaction Technology, Center of Excellence, Bielefeld University, Bielefeld, Germany; 6 Precision Neuroscience & Neuromodulation Program, Massachusetts General Hospital, Harvard Medical School, Boston, MA USA

**Keywords:** IAF-tACS, Postdiction, Rabbit illusion, Space–time illusion

## Abstract

Postdiction is a perceptual phenomenon where the perception of an earlier stimulus is influenced by a later one. This effect is commonly studied using the ‘rabbit illusion’, in which temporally regular, but spatially irregular, stimuli are perceived as equidistant. While previous research has focused on short inter-stimulus intervals (100–200 ms), the role of longer intervals, which may engage late attentional processes, remains unexplored. This study investigates whether postdiction is purely perceptual or also involves attentional mechanisms by using visual stimuli separated by extended intervals. 33 participants (17 females) were assigned to two experimental groups with two different temporal inter-flash intervals (IFI) between stimuli (250 ms: 250-IFI group; 500 ms: 500-IFI). Two stimulation protocols of active transcranial electrical stimulation (tES) and one control condition were tested on the left precuneus/inferior parietal gyrus: (i) transcranial alternating current stimulation (tACS) at the individual alpha frequency (IAF) (IAF-tACS); (ii) transcranial random noise stimulation across the whole alpha band (i.e., 8–12 Hz, Alpha-tRNS) and (iii) a placebo (Sham) stimulation. The postdiction phenomenon was observable in both experimental groups. The participants in the 500-IFI group demonstrated enhanced performance in detecting the illusion during the rabbit illusion task when IAF-tACS was applied. The behavioral results suggest that attentional functions, beyond perceptual ones, play a key role in the postdiction phenomenon.

## Introduction

Time perception is the estimation of the duration of events and the temporal relationship between them (Grondin 2010). The perception of the moment can be influenced by past events, causing a modification of our internal models and temporal expectations about what might happen in the future (Nobre et al. 2007). Indeed, the perception of an event occurring at one moment can alter the perception of a previously presented event (Nadasdy and Shimojo 2010). This phenomenon, known as postdiction (Shimojo 2014), can be observed when successive stimuli are separated in a precise time window (Eagleman and Sejnowski 2000).

 The way an event can alter the perception of something that has already occurred has been investigated using the perceptual effect of the cutaneous rabbit illusion experiment, which is a natural extension of the kappa effect, that is a phenomenon whereby two closely spaced stimuli are perceived as a single touch at a location between the two actual points of contact (Jones and Huang 1982). The rabbit illusion arises when an isochronous sequence of sensory stimuli (temporally regular events) not spatially equidistant (spatially irregular events) are presented to a participant: the isochronous sequence distorts the perceived spatial localization of events so much as to render them equidistant (Geldard and Sherrick 1972; De Pra et al. 2022). The illusory perception of equidistance between stimuli may arise from internal mechanisms prioritizing temporally isochronous events with properties of motion constancy (Rhodes 2018). Illusory phenomena like postdiction are traditionally attributed to low-level perceptual processes, where sensory information is integrated based on temporal proximity. Perception operates according to a probabilistic (or Bayesian) model based on our experience: the inputs arriving from the external world are typically not perceived as isolated, but rather compared to other temporally related stimuli (Goldreich 2007). Thus, the rabbit illusion may instantiate a true temporal illusion based on the timing between stimuli (Dennett and Kinsbourne 1992), providing an ideal paradigm for studying postdiction (Eagleman and Sejnowski 2000; Shimojo 2014). The visual rabbit illusion is a specific form of visuospatial apparent motion, in which spatial localization of stimuli is subjectively shifted based on subsequent input (Grabot et al. 2021). Rather than perceiving three discrete flashes in their actual spatial locations, observers often report the second flash as occurring at an illusory intermediate position, creating the impression of a smooth motion sequence.

The time window or duration over which this mechanism takes place ranges from 80 to 200 ms, and it appears to be primarily triggered by perceptual processing errors (Shimojo 2014). The perception of the position of a visual stimulus can be influenced by motion signals that occur up to ∼80 ms after its appearance (Eagleman and Sejnowski 2000; Eagleman 2008). Indeed, the perception of causality can be conditioned by contextual motion that occurs between 150 and 250 ms after the event, decreasing with a timing greater than 350 ms (Choi and Scholl 2006). The illusion appears to weaken substantially with an interstimulus interval of 300 ms, and disappears entirely at 400 ms (Eimer et al. 2005). This suggests that the illusion is temporally constrained by a perceptual integration window, beyond which higher-order processes are necessary.

Several studies investigated neural correlates of postdiction. An ERP study showed that the visual rabbit illusion involves a cascade mechanism that primarily recruits the occipital regions for low-feature processing of stimuli, followed by the parietal cortex for motion detection, and finally, the frontal cortex for categorization (Stogbauer et al. 2007). A recent study has shown that alpha frequency in the parietal cortex is linked to accuracy in the visual rabbit illusion task (Grabot et al. 2021). Alpha oscillations, which range from 8 to 12 Hz, are the primary spontaneous rhythms in the human brain (Ramsay et al. 2021), and they are most commonly found in the occipital and parietal regions (Lozano-Soldevilla 2018). Alpha oscillations in the parietal cortex are involved in attentional gating, where increased alpha power suppresses irrelevant stimuli (Jensen and Mazaheri 2010). In the context of a visuospatial attention task, alpha power increases in the posterior parietal cortices (Duecker et al. 2017), supporting the inhibition of distracting signals that interfere with information maintained in the temporary buffer (Jensen and Mazaheri 2010). Evidence from neurofeedback, noninvasive brain stimulation, and alpha entrainment suggests that manipulating alpha activity can directly influence attentional tasks performance. Moreover, alpha oscillations are modulated by top-down signals from the frontoparietal networks, which coordinate the selection of relevant sensory input (Peylo et al. 2021).

The perceptual aspect of the rabbit illusion has been thoroughly investigated, but it remains unclear whether the illusion persists with a longer interflash interval (IFI), recruiting visual-attentional resources. Since top-down attentional control engages fronto-parietal networks starting around 250 ms (Schendan and Ganis 2015), the current study modified the IFI of the visual rabbit task to clarify this aspect. Specifically, IFI of 250 ms and 500 ms—250-IFI and 500-IFI conditions, respectively—were designed to differentially engage components of top-down (goal-directed) attentional control. The 250-IFI primarily targets early attentional functions, during which perceptual integration and iconic memory interact with attention modulation, likely reflecting reentrant feedback from higher visual areas (Martínez et al. 2001). In contrast, the 500-IFI is designed to engage late attentional resources and conscious perception, reflecting a global cortical response recruiting working memory, and the reinterpretation of previous sensory input (Cul et al. 2007). Indeed, after about 300 ms, the vivid details held in iconic memory begin to fade rapidly, and the information is transferred into a more stable but limited-capacity memory system, such as working memory (Bradley-Garcia et al. 2023).

In addition, transcranial electrical stimulation (tES) was employed to modulate the task performance: tES is a non-invasive brain stimulation technique that can be delivered concurrently with the execution of a specific task or training to modulate brain oscillatory activity and change the related performance (Lazzaro et al. 2022). Alpha-frequency tES has already been used to effectively modulate specific brain regions involved in attentional tasks (Kemmerer et al. 2022). The protocols used in the present study included: (i) a transcranial alternating current stimulation (tACS) at the individual alpha frequency (IAF-tACS); (ii) a novel transcranial random noise stimulation (tRNS) delivering current within the alpha band (i.e., 8–12 Hz, Alpha-tRNS); (iii) and a placebo (Sham) stimulation.

The primary aim of the study was to examine the role of top-down resources on postdiction, and, more specifically, (a) whether the phenomenon can be elicited by longer IFI involving early and late attentional processes; (b) whether tES has the potential to modulate the functioning of brain regions associated with attentional abilities.

## Materials and methods

### Participants

G-Power was used to calculate the appropriate sample size, running an a-priori analysis of variance (ANOVA, within-between factors interaction). Input parameters of the simulation were: 3 tES conditions (i.e., IAF-tACS; Sham; Alpha-tRNS); 2 groups (i.e., 250-IFI; 500-IFI); effect size f: 0.25; alpha: 0.05; 1-beta error probability: 0.85 (Faul et al. 2007). Analysis showed that a total sample of 32 participants was sufficient to achieve a statistical actual power of 0.85 and discriminate significant differences between different conditions (Noncentrality parameter λ: 12; Critical F: 3.15; degrees of freedom: 2,60). Thirty-three right-handed subjects (25.4 ± 3.6 years old; 17 F) were recruited at the University of Siena, Italy. Participants were excluded if they a) had a history of seizures, b) had a severe and uncorrectable visual impairment, c) were taking any medication that could alter cortical excitability and d) had a diagnosis of any neurological or psychiatric disorder. Participants signed an informed consent form before effective participation. The study protocol adhered to the tenets of the Helsinki Declaration and was approved by the local ethical committee (protocol code: Brainsight 21/24).

### Stimulation montage

The tES montage was optimized using SimNIBS software and the “Ernie” head model (Thielscher et al. 2015). The left precuneus and the left inferior parietal gyrus were identified as the target stimulation sites, as they are involved in the perception of the rabbit illusion (Grabot et al. 2021). A 4-electrode montage was calculated (Fig. 2A, B), and the electrodes were placed at TP7 (1500 µA), at CP5 (2000 µA), at P1 (2000 µA) and at PZ (1500 µA) positions in the 10–10 electrode system for EEG. tES was delivered using a Starstim 32-channel neurostimulator from a PC via USB (Windows 11 operating system Microsoft Corporation, Redmond, Washington—USA) and controlled with NIC2.0 software (Neuroelectrics®, Barcelona, Spain). Pistim Ag/AgCl electrodes (contact area π cm^2^ ≈ 3.14 cm^2^) were used (Neuroelectrics^®^, Barcelona, Spain) and conductive gel was applied and electrode–skin impedances were continuously monitored and kept below 10 kΩ. In the IAF-tACS condition, each participant received a sinusoidal stimulation at their individual alpha frequency (IAF) with fixed inter-channel phases (P1/Pz: 0°; CP5/TP7: 180°). Sham stimulation was administered using a standard protocol, in which the current ramps up and down over a 30-s interval at the beginning and end of the session to mimic the somatosensory sensations of active stimulation (Ambrus et al. 2012). For the first time, we also implemented an Alpha-tRNS protocol, with the aim of enhancing alpha activity in the parietal cortex through a randomized stimulation profile constrained within the alpha frequency band (8–12 Hz), zero-mean noise with no inter-electrode phase-locking.

### Experimental design

Participants were seated in a chair with an armrest and a backrest. At the beginning of the experiment, the attentive matrices test (AMT) (Bianchi and Dai Prà, 2008) and the trail-making test part A and B (TMT A and TMT B) (Amodio et al. 2002) were administered. Specifically, the AMT assesses visuospatial attention and requires participants to search for specific numbers between multiple distractors as quickly as possible. In TMT A, participants must connect a set of 25 numbered dots as quickly as possible. On the other hand, TMT B consists of thirteen numbered dots (from 1 to 13) and twelve lettered dots (from A to L), and the participants must connect numbers and letters alternately (1-A-2-B, and so on). The time difference between the B and A parts of the test (TMT B-A) is a valid measure of the subjects’ executive function and processing speed: a longer TMT B-A time indicates poorer executive function and slower processing speed (Periáñez et al. 2007).

**Fig. 1:**
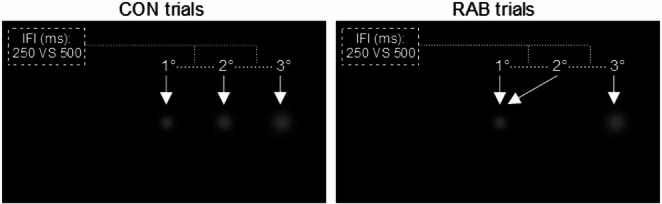
A sequence of three isochronous flashes (2-D gaussian blobs) was presented. In the control trials (CON), the second flash was in an intermediate location between the first and third. In the test trials (RAB), the second flash was at the same location as the first. Participants were instructed to press number 1 on the keyboard if they perceived the second flash in an intermediate position between the first and the third. Participants were assigned to either a 250-IFI or a 500-IFI group and received tES during the task

Subsequently, the participants carried out the rabbit task that was based on Grabot et al. (2021). The distance between the eyes and the center of a monitor was 40 cm, in which an isochronous sequence of three white flashes (M-scaled 2D Gaussian blobs with a peak contrast of 0.4) was presented on a black screen (Fig. 1). The sequence of flashes was presented horizontally, along the midline, going outward relative to the fixation dot, which was centered on the screen. The three flashes fell exactly at 15.1°, 18.5°, and 21.8° of the visual angle and were presented for 50 ms each. To correct the increasing distance of the flashes from the fovea, the diameter of each flash increased with eccentricity, with an effective flash degree of 2.5°, 3°, and 3.5° of the participant’s visual angle, respectively (Duncan and Boynton 2003; Grabot et al. 2021).

Two types of sequences were randomly tested: the control trials (CON), in which visual flashes were presented isochronously and equidistant (regularly spaced in time and space); and the rabbit trials (RAB), in which the sequence was isochronous, but the second flash in the sequence was presented in the same position as the first (thus, flashes were regularly spaced in time but not in space). In each trial, participants had to respond to the question: "Was the second flash in an intermediate position between the first and third?" by pressing the number 1 for YES or the number 2 for NO response on a keyboard. The percentage of the correct responses in both CON and RAB trials was calculated. From this point onward, the term “correct responses” refers to the physically accurate location of the second flash. Although in RAB trials, this term could be conceptually problematic—since the second stimulus is illusory and its perception inherently subjective—we adopt it to maintain a consistent analytical distinction between responses that align with the actual physical location and those that do not. Specifically, in CON trials, the percentage of correct responses reflects baseline perceptual accuracy, whereas in RAB trials it indicates that the illusion was not perceived, and the participant correctly identified the veridical (non-illusory) location of the second stimulus. The time required to generate the response was not recorded, as there was no specific request for a prompt response. The inter-trial interval was randomly assigned between 0.8 and 1.3 s following each response. Participants were assigned either to the 250 ms IFI (250-IFI group; 16 participants; 25.4 ± 3.3; 7 females) or to the 500 ms IFI group (500-IFI group; 17 participants; 25.5 ± 4.0; 8 females).

**Fig. 2:**
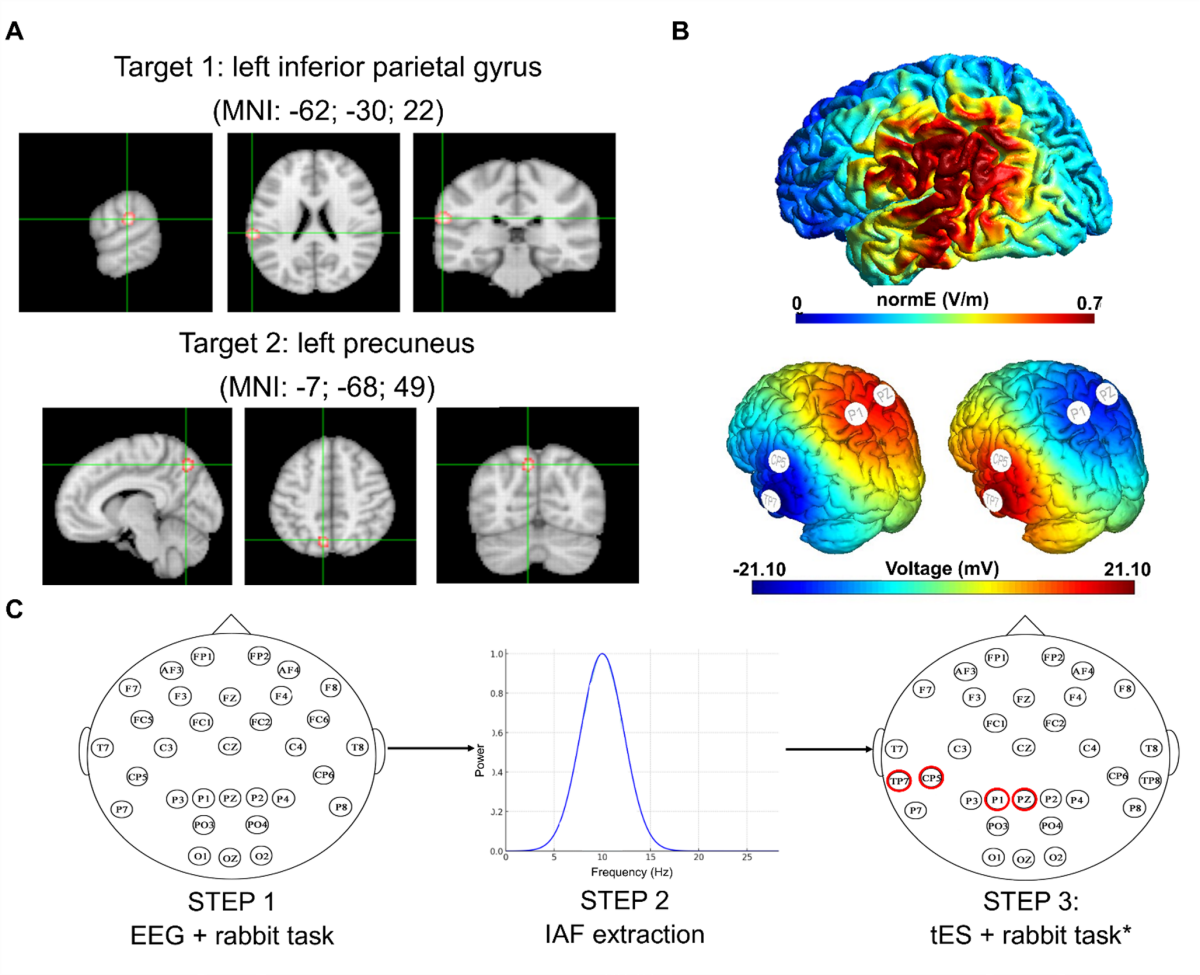
**A** The left inferior parietal gyrus (MNI: − 62; − 30; 22) and the left precuneus (MNI: − 7; − 68; 49) were selected as stimulation targets based on prior work (Grabot et al. 2021).** B** The 4-electrode stimulation montage (TP7: 1500 µA; CP5: 2000 µA; P1: 2000 µA; and PZ: 1500 µA) was modeled to maximize the field strength over the left inferior parietal gyrus and the precuneus. We show an estimation of the electric field magnitude (normE; V/m) on the cortical surface from SimNIBS modeling and the corresponding per-channel voltage (mV) estimated with the NIC 2.0 software with in-phases (Pz/P1 at 0°) and antiphases (TP7/CP5 at 180°). **C** Participants underwent 32-channel EEG recording during a baseline session of the rabbit task. The individual alpha frequency peak (IAF) was extracted, and participants were stimulated for three random tES sessions while performing the rabbit illusion task. *stimulation sessions consisted of an IAF-tACS, an Alpha-tRNS, and a Sham protocol (red circles: electrode stimulation sites)

All participants underwent four rabbit task sessions. Initially, electroencephalographic data (EEG) was recorded using a Starstim 32-channel device (Neuroelectrics®, Barcelona, Spain) during a baseline session of the rabbit task (40 CON and 80 RAB trials, randomly interleaved). At the end of the baseline task, the individual alpha frequency (IAF) peak was extracted using the Darbeliai script of the EEGLAB open-source toolbox (Delorme and Makeig 2004). This script automates the identification of the IAF peak by applying a power spectral density (PSD) analysis to the EEG data. Specifically, it computes the frequency spectrum for each participant, typically utilizing the Fast Fourier Transform (FFT), and identifies the dominant peak within the alpha band (8–12 Hz). This approach ensures a data-driven and individualized determination of the IAF value, which can then be used for personalized neurostimulation protocols such as IAF-tACS. Then, each participant was stimulated for three consecutive tES sessions in randomized order: Alpha-tRNS (8–12 Hz), IAF-tACS, and Sham (Fig. 2C) were administered throughout the entire execution of the rabbit task (60 CON and 120 RAB trials in each session; mean duration: 11 min). Both participants and experimenters were blinded to the stimulation condition being administered.

### Statistical analysis

Statistical analyses were conducted using SPSS version 16 (SPSS Inc., Chicago, IL, USA) on the percentages of correct responses obtained on both the RAB and CON trials. For all tests, the significance level was set at α = 0.05.

- Multivariate test was conducted to assess differences in task correct responses across trial types (RAB and CON, within-subjects factor) at baseline for both 250‑IFI and 500‑IFI groups.
- For each IFI group (250‑IFI and 500‑IFI), a repeated measures ANOVA (RM-ANOVA) was performed to compare the percentage of correct responses across trial type (RAB vs. CON, within-subjects factor) and tES sessions (Sham, IAF‑tACS, Alpha‑tRNS, within-subjects factor).
- Pearson’s correlation analyses were performed between participants’ IAF value and behavioral measures, including TMT-B and TMT B-A completion time and percentage of correct responses in the rabbit illusion task (RAB and CON trials).

## Results

All participants tolerated the tES procedure well, reporting only mild numbness beneath the stimulation site. One participant from the 500‑IFI group was excluded from further analyses due to achieving 0% correct responses on the RAB trials. Consequently, statistical analyses were conducted on data from 32 participants (250‑IFI group: 16 participants, 25.4 ± 3.3 years, 7 females; 500‑IFI group: 16 participants, 25.8 ± 3.8 years, 8 females). Table 1 presents the mean percentages and standard errors (SE) of correct responses for the rabbit task, separately for RAB and CON trials within each IFI group, as well as correct responses across all stimulation modalities (IAF‑tACS, Alpha‑tRNS, and Sham).

**Table 1:** Rabbit task correct responses. The percentage of correct responses (mean and *standard error*) for the CON and RAB trials in each stimulation session (IAF-tACS, Alpha-tRNS, and Sham) and for each group (500-IFI and 250-IFI) is shown

500-IFI GROUP								
Session	BASELINE		Sham		IAF-tACS		Alpha-tRNS	
Trials	CON	RAB	CON	RAB	CON	RAB	CON	RAB
Percentage of correct responses (mean)	88.32	50.72	87.84	52.02	83.33	57.81	87.76	55.04
*Standard error*	*1.82*	*3.92*	*1.89*	*4.82*	*2.44*	*4.78*	*2.06*	*5.07*

250-IFI GROUP								
Session	BASELINE		Sham		IAF-tACS		Alpha-tRNS	
Trials	CON	RAB	CON	RAB	CON	RAB	CON	RAB
Percentage of correct responses (mean)	84.17	51.98	80.42	47.29	83.65	43.96	80.52	45.78
*Standard error*	*2.80*	*4.91*	*2.47*	*4.64*	*2.53*	*4.23*	*2.78*	*4.70*

**Fig. 3:**
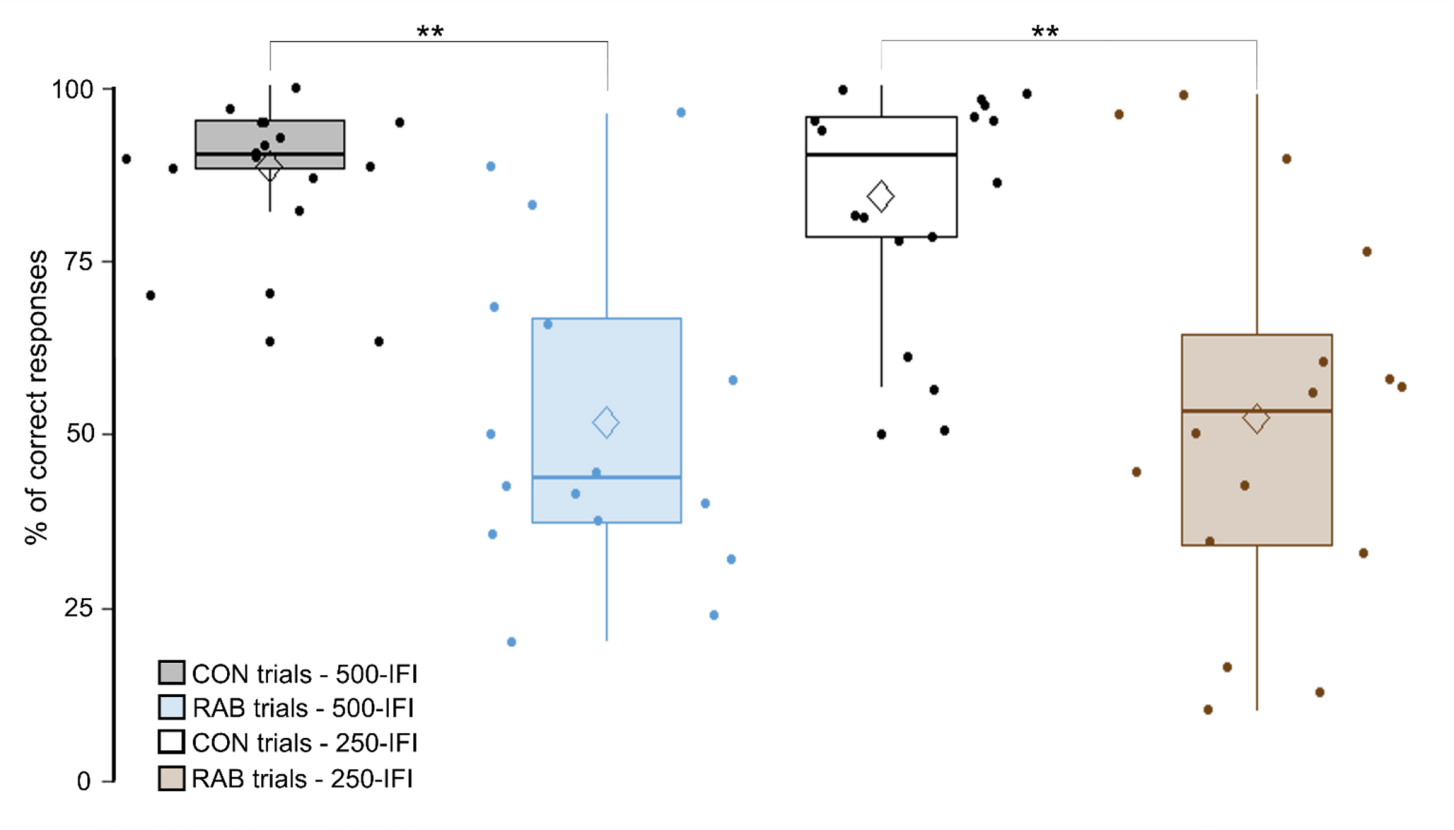
Graph of the rabbit task correct responses at baseline. The analysis shows a significantly higher percentage of correct responses on CON trials than on RAB trials (** *p* < .001). No difference was found between the 250-IFI and 500-IFI groups

The multivariate test on task correct responses (see Fig. 3) revealed a significant main effect of trial type at baseline for both the 250-IFI group (F_(1,30)_ = 16.21, *p* < 0.001), and the 500-IFI group, (F_(1,30)_ = 36.92, *p* < 0.001). Bonferroni-adjusted pairwise comparisons indicated that both the 250‑IFI and 500‑IFI groups showed significant higher correct responses on CON trials compared to RAB trials (*p* < 0.001).

**Fig. 4:**
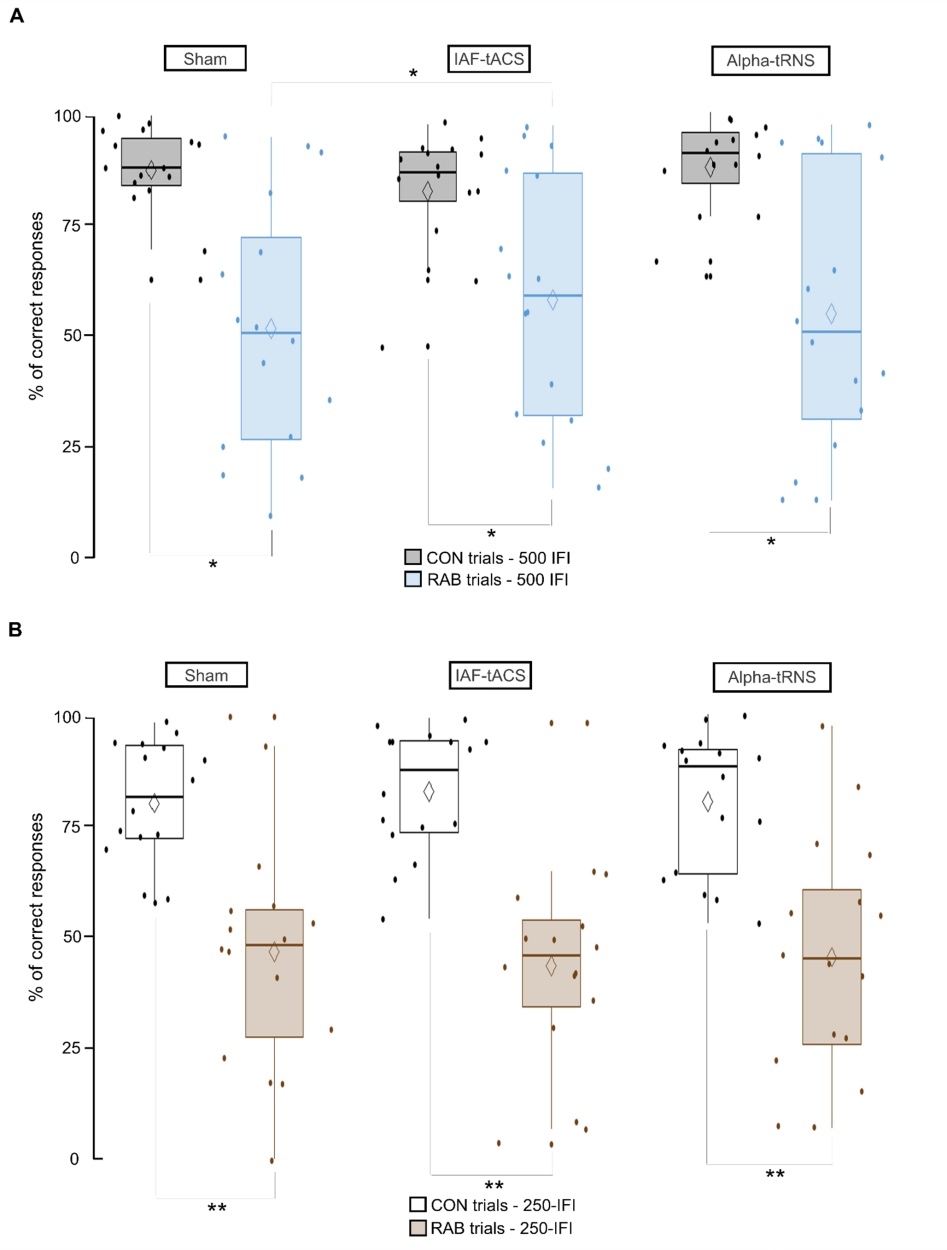
The graph depicts the rabbit task correct responses during the stimulation sessions. The percentage of correct responses in the 500-IFI **A** and 250-IFI **B** trials is shown. A significant main effect of trial type was observed. The percentage of correct responses was higher on the CON trials than the RAB trials in both the 500-IFI and 250-IFI groups. Moreover, the 500-IFI group exhibited a significant higher percentage of correct responses on the RAB trials during IAF-tACS compared to the Sham session (*p < .05; **  *p* < .001)

The RM-ANOVA analyzing correct responses on the RAB and CON trials across stimulation sessions (Fig. 4) revealed a significant effect of trial type for both the 500-IFI (F_(1,15)_ = 12.65; *p* = 0.003) and the 250-IFI groups (F_(1,15)_ = 20.57; *p* < 0.001). Bonferroni-adjusted pairwise comparisons indicated an higher percentage of correct responses in CON trials than in RAB trials (*p < 0.05*  in the 500-IFI group and *p* < 0.001 in the 250-IFI group). Additionally, a significant interaction between trial type and stimulation session was observed in the 500-IFI group (F_(2,14) _= 4.24; *p* = 0.036), but not in the 250-IFI group (F_(2,14)_ = 1.195; *p* = 0.332). Post hoc Bonferroni-corrected pairwise comparisons indicated that, in the 500-IFI group, the percentage of correct responses in RAB trials was significantly higher during the IAF-tACS session compared with the Sham session (*p* = 0.028). All other comparisons did not reach significance threshold Specifically, for CON trials: Sham versus IAF-tACS (*p* = 0.204), Sham versus Alpha-tRNS (*p* = 1.00), and IAF-tACS versus Alpha-tRNS (*p* = 0.096). For RAB trials: Sham versus IAF-tACS (*p* = 0.021), Sham versus Alpha-tRNS (*p* = 0.610), and IAF-tACS versus Alpha-tRNS (*p* = 0.810). Although no significant interaction between stimulation session and trial type emerged in the 250-IFI group, statistics of all pairwise comparisons are reported for completeness. For CON trials: Sham versus IAF-tACS (*p* = 0.337), Sham versus Alpha-tRNS (*p* = 1.00), and IAF-tACS versus Alpha-tRNS (*p* = 0.302). For RAB trials: Sham versus IAF-tACS (*p* = 0.784), Sham versus Alpha-tRNS (*p* = 1.00), and IAF-tACS versus Alpha-tRNS (*p* = 1.00).

Results revealed significant negative correlations between IAF value and completion time on both the TMT_B (r = − 0.405, *p* = 0.022) and the TMT_B–A (r = − 0.538, *p* = 0.001). Furthermore, IAF value was negatively correlated with the percentage of correct responses in the RAB trials during the Sham (r = − 0.448, *p* = 0.010), IAF-tACS (r = − 0.361, *p* = 0.042), and Alpha-tRNS (r = − 0.422, *p* = 0.016) sessions. No significant correlations were observed between IAF value and performance on the CON trials in the Sham, IAF-tACS, and Alpha-tRNS sessions (r = − 0.131, *p* = 0.474; r = 0.015, *p* = 0.936; r = − 0.045, *p* = 0.806, respectively). No statistically significant correlation was observed between the number of RAB/CON trials and AMT performance (*p*>.05).

## Discussion

Temporal illusion tasks can be used to investigate the phenomenon of postdiction (Shimojo 2014), which is based on the concept that an event can influence how sensory inputs are retrospectively reconstructed in memory, contributing to generating temporal illusions (Dennett and Kinsbourne 1992). The rabbit illusion creates a “phantom” cutaneous perception that connects points on the skin that are actually being touched (Geldard and Sherrick 1972). The task induces an illusory temporal perception, and it has been extensively used to investigate postdiction in the tactile domain (Blankenburg et al. 2006; Goldreich and Tong 2013). This phenomenon has been recently explored in other sensory modalities using auditory (Stiles et al. 2018) or visual stimuli (Khuu et al. 2011; Grabot et al. 2021). The literature reports that there is a short time window of less than a quarter of a second within the presentation of a stimulus that can influence the perception of another previously presented (Yamada et al. 2015).

In the current study, a visual rabbit task with a longer IFI was also employed to investigate the role of early (Mangun and Hillyard 1991; Martínez et al. 2001) and late (Bradley-Garcia et al. 2023) attentional top-down attentional components in the context of postdiction. The percentage of correct responses on the CON and RAB trials (with respective general means of 86.6 and 50.5) was comparable to previously reported findings (Grabot et al. 2021), demonstrating that postdiction phenomenon may also be evoked with a longer IFI, at least in the visual domain. The percentage of correct responses on the CON trials with stimuli separated by a regular spacing was similar in both experimental 250-IFI and 500-IFI groups and in all stimulation sessions. This finding suggests that, under the specific conditions of our task, tACS did not significantly alter the ability to process or respond to these regularly timed stimuli. However, these results only pertain to the attentional demands inherent in our experimental paradigm and cannot be generalized to all aspects of attentional function or to different task contexts.

The mean of correct responses on the RAB trials does not align with the results of a previous study that observed no illusion effect when the interstimulus interval exceeded 350 ms (Khuu et al. 2010). One potential explanation for the discrepancies between our findings and those reported by Khuu et al. (2010) lies in the differences in experimental paradigms and task parameters. While Khuu et al. employed a design that may not have fully captured the temporal integration processes required to induce the illusion at longer IFI, our study incorporated a more sensitive measure of perceptual integration. Specifically, our task design allowed for a broader range of IFI and included conditions that enhanced the temporal predictability of the stimuli, which might facilitate the illusion effect even at extended intervals. Additionally, small variations in stimulus presentation—such as differences in the spatial configuration of stimuli, the intensity of visual cues, and the response timing protocols—could have contributed to a greater propensity for illusion perception in our study. Further experimentation may be conducted to ascertain the temporal parameters associated with the dissolution of the illusion. However, our outcome provides new insights into the postdiction, demonstrating that the phenomenon can be elicited by a larger interval between stimuli, which loads on attentional resources with a possible link to retro cueing (Sergent et al. 2013) and “long-lasting effects” of postdiction (Herzog et al. 2020).

Considering the difference between the stimulation protocols, the RAB trials results indicate that the random stimulation of all alpha bands (Alpha-tRNS) does not affect percentage of the correct responses in either the two IFI groups. In contrast, responses on the RAB trials exhibited significant modulation by tACS-IAF. However, the nature of this modulation differed in the 250-IFI versus 500-IFI groups. In the 250-IFI group, the percentage of correct responses on the RAB trials was not influenced by the type of stimulation protocol employed, and equivalent performance was observed in Sham, IAF-tACS, and Alpha-tRNS sessions. Conversely, participants in the 500-IFI group showed an increase in correct responses on the RAB trials, thus a decrease in illusory reports, during the IAF-tACS compared to the Sham stimulation.

Current results indicate that the exogenous individual alpha rhythm provided by the IAF-tACS may have increased the alpha coherence and the associated attentional top-down processing of visuospatial stimuli, resulting in an advantage in the correct localization of the stimulus on the rabbit trials. These findings provide further evidence that alpha coherence and top-down attention is a mechanism by which the fronto-parietal network might control spatial attention (van Schouwenburg et al. 2017). Thus, illusion detection may extend beyond perceptual processing and can be influenced by the recruitment of late top-down attentive components. Previous studies linked cognitive performance to endogenous modulations of oscillatory neural activity in the IAF (Klimesch 2012). Indeed, the IAF appears to predict correct responses percentage not only in perceptual (Cecere et al. 2015; Samaha and Postle 2015) but also in attentional tasks (Klimesch et al. 2006; Bornkessel-Schlesewsky et al. 2022).

The observation that the IAF-tACS did not influence the correct responses on the RAB trials in the 250-IFI group may be related to the concept that the stimuli detection is linked to the alpha band oscillation peaks. Perception operates in cycles with high and low periods of excitability (VanRullen et al. 2011). The illusory percept is most commonly identified when the IFI delay aligns with the period of the oscillatory impulse response function reverberating in parietal alpha oscillations (Gulbinaite et al. 2017). It is possible that 250 ms may be a suboptimal timing to fully synchronize with natural alpha oscillation, as evidenced by the observation of a trend of lower correct responses in 250-IFI compared to 500-IFI (percentage of the correct responses on RAB trials considering in stimulation conditions: 45.67% and 56.27% for 250-IFI and 500-IFI, respectively). Otherwise, it was postulated that each alpha peak may correspond to each single trial flash in the 500-IFI group and each flash corresponding every ~ 5 peaks of alpha (IAF mean: 10.53 ± 0.7 and 10.15 ± 0.6 for 250-IFI and 500-IFI group respectively). This natural synchronization between flash lags and natural alpha frequency peaks may be further strengthened by the IAF-tACS and leads to a higher correct responses percentage in the task. Another possible explanation for this selective effect is that IAF-tACS may have enhanced not only top-down attentional processes but also working memory functions, thereby boosting both executive functions during the task execution. Indeed, according to our hypothesis, these cognitive resources are strongly recruited during the 500-IFI task, where perceptual integration likely relies on active maintenance of sensory input (Andersen and Buneo 2002; Cul et al. 2007; Bradley-Garcia et al. 2023). Future research should consider the stimulation of alternative nodes of the visuospatial network, which are linked to perceptual processing and using different IFI or connected to early attentive functions. Potential areas for stimulation include the primary visual cortex (V1) or the extrastriate visual cortex (Ungerleider 2000; Treue 2003) which are recruited during the task execution (Grabot et al. 2021).

Lastly, the correlation analysis shows two interesting results. First, subjects with a higher IAF value performed the TMT-B faster than participants with a low IAF value. This result seems to align with what has been reported in literature, where IAF value positively correlates with processing speed (Ociepka et al. 2022). Second, IAF value is inversely correlated with the percentage of correct responses in the RAB trials. This finding is consistent with a result presented in literature, suggesting that a lower IAF value is associated with better detection of moving visual stimuli (Howard et al. 2017). One possible interpretation is that lower IAF value reflects a slower internal sampling rate, which allows for more stable sensory integration and less susceptibility to postdictive distortions. In this framework, participants with lower IAF value may rely more on veridical sensory information, rather than integrating stimuli into a single motion trajectory based on temporal regularities. As a result, they are more likely to correctly detect the non-illusory location of the second flash in the RAB trials. Altogether, this finding supports the importance of individual differences in temporal processing and provides a neurophysiological basis for the subjective nature of the rabbit illusion. Further research is needed to clarify the specific contributions of alpha frequency to individual differences in visuospatial abilities and its potential applications in cognitive neuroscience and clinical practice.

### Limitations of the study

The main limitation of the study is the small sample size in each IFI group. Finally, stimulation of the occipital brain area associated with perceptual processing and using a task with a different IFI could lead to differences in the percentage of the correct responses between the groups and should be tested in a future investigation.

## Conclusions

The phenomenon of postdiction appears to be modulated to both perceptual and attentional mechanisms. In our study, IAF-tACS facilitates top-down control processes, which may reduce susceptibility to the visual illusion of postdiction, suggesting a potential role of intrinsic oscillatory dynamics in modulating perceptual integration over time.

## Data Availability

Data are available upon reasonable request, and can be accessed by contacting corresponding author.
